# Effects of heterogeneous SPS measures on agricultural growth: Evidence from China

**DOI:** 10.1371/journal.pone.0266904

**Published:** 2022-05-10

**Authors:** Xuemei Liu, Wei Chen, William H. Meyers, Yinguo Dong

**Affiliations:** 1 Alibaba Business School, Hangzhou Normal University, Hangzhou, China; 2 School of Social and Public Administration, East China University of Science and Technology, Shanghai, China; 3 Division of Applied Social Sciences, University of Missouri, Columbia, Missouri, United States of America; 4 School of Business, East China University of Science and Technology, Shanghai, China; International Centre for Integrated Mountain Development (ICIMOD), Kathmandu, Nepal, NEPAL

## Abstract

In order to explore the sustainable growth of Chinese agriculture, this paper assesses how the heterogeneous SPS measures affect China’s growth margins and quality upgrading in the agricultural sector. We estimate improved gravity-model that exploit the cross-country differences in SPS measures over the period from 2000 to 2014. Our findings show that the heterogeneous SPS measures restrict the intensive margin and extensive margin, but significantly promote the product quality-even with no significant effect on price index. Conditional on quality upgrading, the heterogeneous SPS measures decrease the extent of quality upgrading. On one hand, because of cross-country differences in SPS standards, standards from developed countries have less effective trade effect on China’s agri-products export quality upgrading; On the other hand, because of cross-firm differences in the ability to deal with SPS standards, the laggards in China have the higher probability to switch to lower entrance barrier countries. Therefore, it is possible for China to trap in the low-quality agricultural growth in the long term.

## Introduction

How international quality standards affect the sustainable growth is a hot topic in the global agricultural area. As many problems have been raised in the production and processing of agri-food, such as footborne pathogens, the extensive use of antibiotics, pesticides and fertilizer, industrial pollutants, new allergens caused by genetically engineered material [1], the sustainable growth of agriculture has caught the attentions of governments, researchers and the public. Meanwhile, the heterogeneous product quality has become the current frontier field in international trade [2]. Especially, when focusing on the agri-products area, assumptions about homogeneity in product quality has been broken and more researchers believed that cross-country and cross-firm differences in agri-products’ quality which affect the consumer’s preference [3, 4]. In the past, the consumers’ interest involves freshness, expected appearance, taste and texture, etc. But now, consumers also care about the appearance of the production processes and their impact on the environment. The high-quality and cost-effective agri-products are more accessible in the consumer markets. As a result, the impetus of sustainable growth and international competitiveness of agri-products have switched from price-driven to quality-driven.

**Fig 1:**
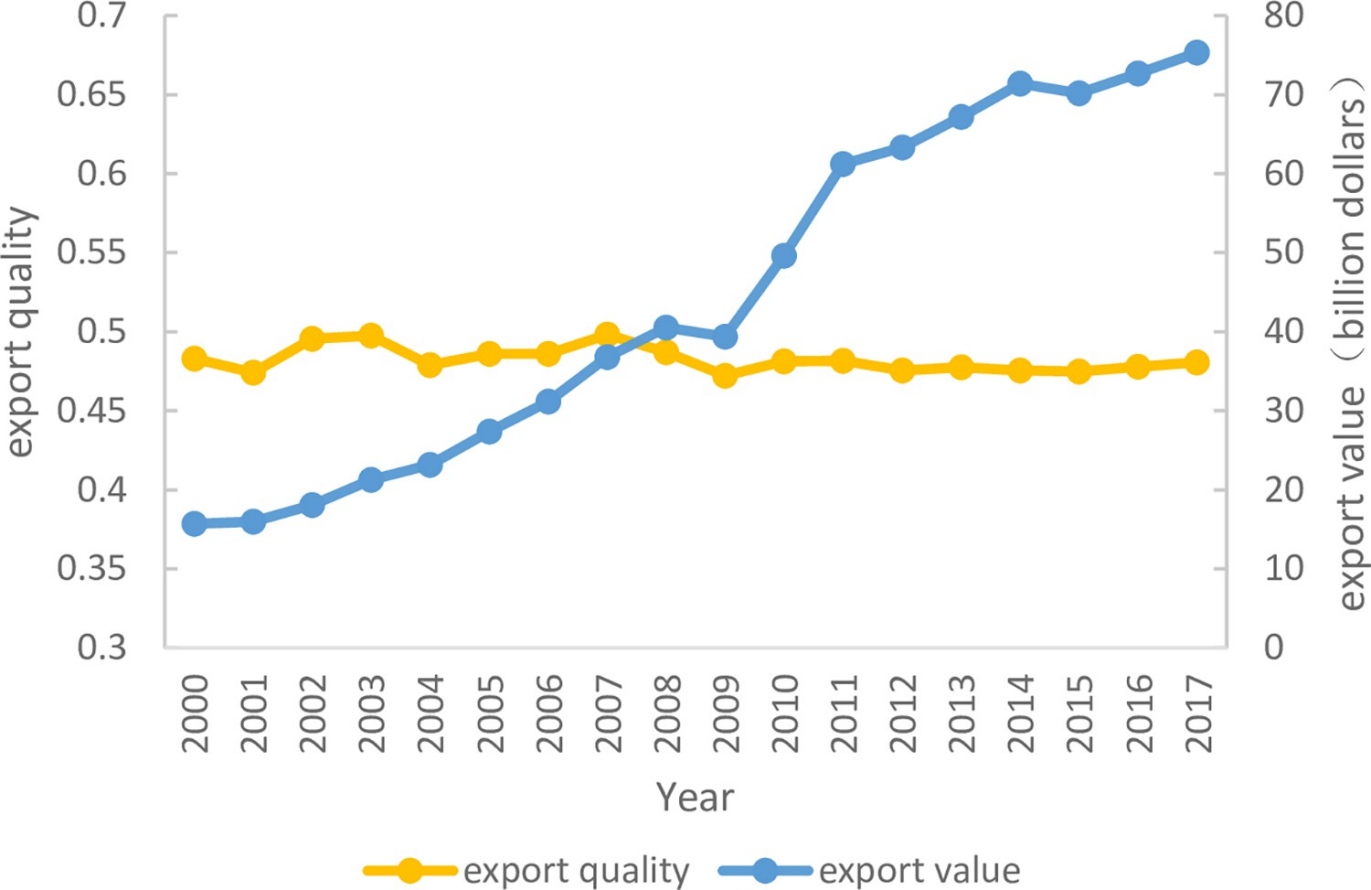
Export value and export quality of China’s agri-products (2000–2017).

Agri-products export is an indispensable part of sustainable agricultural growth. It not only adjust surplus and ensure supply, but also exert comparative advantages and optimize domestic resource allocation [5]. However, there are many sides need to be improved in China. Firstly, there is a higher variety similarity of import and export. For example, based on the UN Comtrade database, there are 532 similar varieties on HS6 digital level in 2017, and they have taken 75% of the whole export varieties and 93% of the export values. Secondly, the quality level of import and export is quite different. For example, Brazil and the United States, as the agricultural powerful and high-quality countries, have taken 20.53% and 18.81% of China’s import separately. But China’s exporting destinations are mostly focused on Association of Southeast Asian Nations (ASEAN) and some other developing countries, which provide agri-products in low and medium quality. Thirdly, if China over-reliances on foreign agri-products, it will induce series problems in national security and trade balance in the future. Finally, by using the quality measurement method measure China’s export quality [6], the result (as shown in the Fig 1) illustrates China’s export quality (0.45) is uncoordinated with its international market position as the first agricultural production country. Obviously, in China, the quality upgrading is not yet the impetus of sustainable growth.

**Fig 2:**
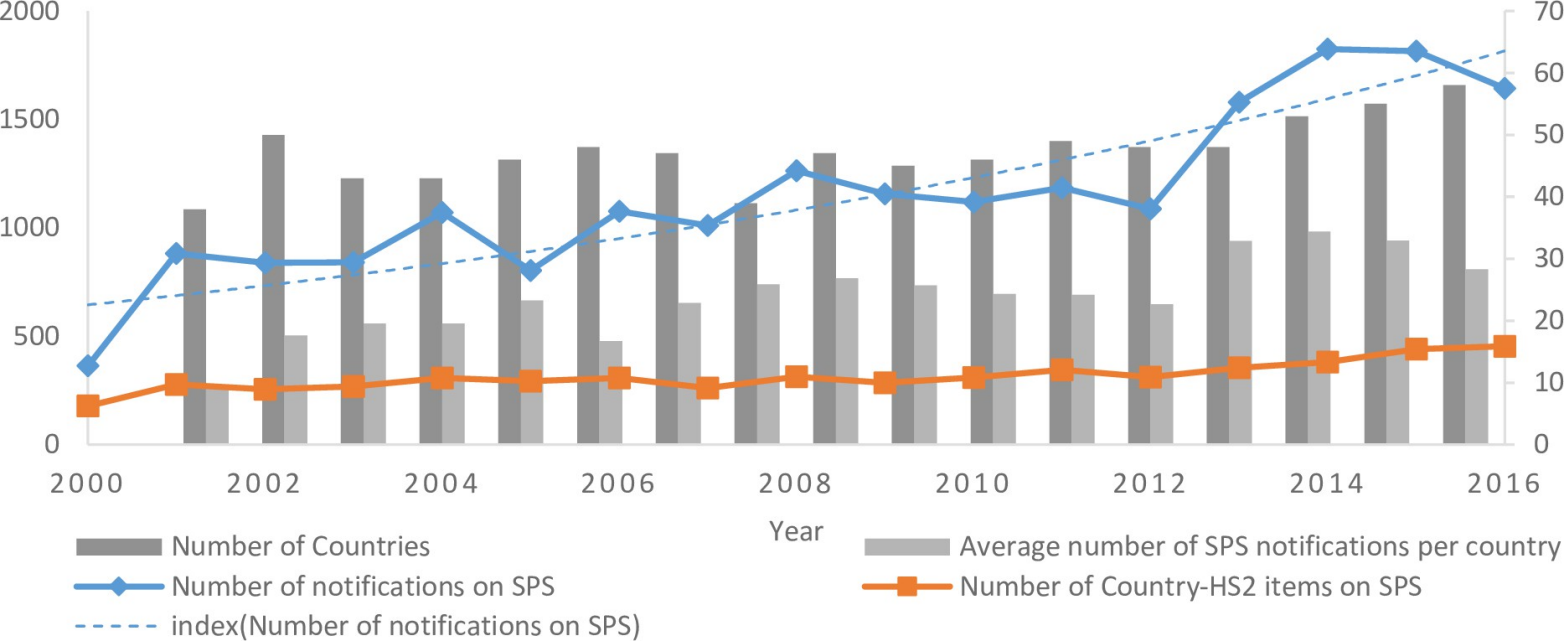
Notification number of SPS measures (2000–2016). Notes: Data from WTO-SPS.

As tariff has been gradually lowered over the past few decades, Sanitary and phytosanitary (SPS) measures, as the important quality standards, are precisely imposed to protect quality safety and keep agri-products trade in sustainable development. SPS measures have became the key focus for increased market access of international agricultural markets. However, it is not easy to know whether the international SPS measures play as catalysts or barriers. On one hand, in order to satisfy consumers’ quality preferences, SPS measures ensure the safety and quality. On the other hand, they might help the domestic producers keep far away from the international competition [7]. There are three features of SPS measures to note. First, based on the WTO-SPS database, Fig 2 presents the full sample of SPS notifications that includes the number of SPS measures, country-HS2 items on SPS, the number of countries on SPS and average number of SPS notifications per country. All of the indexes have been increasing over the period analyzed. The accumulated number of notifications during the period 2000–2016 are more than 19,000. The formulation and frequent replacement of SPS measures increases the compliance costs, which will inhibit the export growth and the variety expansion [8].

Second, the heterogeneity of SPS measures is the most special feature because of the cross-country and cross-variety differences in SPS measures. For instance, when talking about the minimum and maximum residue levels (MRLs), the report of EPCARD shows that Argentina, Australia, Mexico, and the United States implement the most stringent standards. The MRLs standards of Wheat (0.05, 0.5) is more stringent than Banana (3, 3) in EU and Japan respectively [4]. Meanwhile, SPS measures have raised the doubt of the implement of SPS measures. From 1995 to 2017, the number of specific trade concerns (STCs) regarding SPS measures among WTO-SPS committee members reached 434. United States, EU, Japan, Philippine and Brazil are not only the top-5 SPS implementing countries, but also getting more STCs.

Third, the threshold effect of SPS measures is nonegligible, even though SPS measures have improved the quality standards and increased the transparency of the production process under the non-discriminatory principle. Especially, SPS measures have become the top barriers to poor-quality countries’ argi-products export to high-standard countries [9–11]. The threshold effect is especially pronounced for South-North trade, but not for exports to the South [4].

Therefore, it is necessary to study the export growth (growth margin and quality upgrading) by focusing on the heterogeneous SPS measures. Many articles have thoroughly proved the trade effects of SPS measures on export growth. Some insist SPS measure are trade catalyst (e.g., demand-enhancing effect, welfare-increasing effect,) [12, 13] and some stick them are trade barriers (e.g., cost-increasing effect, competition reducing, protect domestic market effect) [3, 7, 14]. Meanwhile, there are few articles talked the relationship between SPS measures and quality upgrading [15]. Considering the heterogeneous consumers’ quality preferences in different destinations, some papers insist the per capital GDP of the destinations as an important quality-affecting factor at the national level [16]. When explaining the firm-level export quality, there are a few studies discussed the productivity [17], path dependence [18], and exchange rate [19]. In order to analyze the relationship between market similarity and corporate financing constraints, Tan et al. [20] integrate product quality into a multi-destination trade model and explain the export firm’s quality difference from the perspective of financing constraints. A limitation in these literatures is the almost exclusive focus on trade effects of SPS measures while ignoring the heterogeneity of SPS measures. Until now, Fontagné & Orefice [21] is the first to present the mechanism of heterogeneous technical barriers to trade (TBTs), which are the barriers mainly implemented in manufacture sectors. By combining TBTs with a multi-destination trade model, Fontagné & Orefice find stringent TBTs drive the average firm out of the market with a magnified effect for multi-destination players, who are encouraged to redirect their exports to other destinations (free for TBT concerns). However, the study from the perspective of heterogeneous SPS measures effects on the quality upgrading is still missing.

Our paper tries to evaluate heterogeneous SPS measures in the multi-destination context and assesses how heterogeneous SPS measures affect China’s export margins and quality upgrading. Our work extends the existing literature in two ways. First, in terms of the research perspective, we focus on quality upgrading and analyze the dynamic effects of heterogeneous SPS measures by weighing the compliance costs and switch costs. Second, further analyses are conducted on the heterogeneity across country different routes and across firm different trade routes.

The rest of the paper proceeds as follows. Section 2 discusses the heterogeneity of the SPS measures. The export facts of China’s agri-product are illustrated in Section 3. Section 4 builds the empirical model and describes the data. We report and explain estimation results in Section 5. Section 6 discusses our main findings and offers policy recommendations. At last, Section 7 summarizes the conclusions.

## Discussion on the heterogeneity of the SPS measures

The heterogeneity of the implementation of SPS measures at different HS digital level is from the UNCTAD-Trains database, which involves the duality of SPS measures, the pertinacity of the destination country standards and the diversity of categories.

### The duality of SPS measures

**Fig 3:**
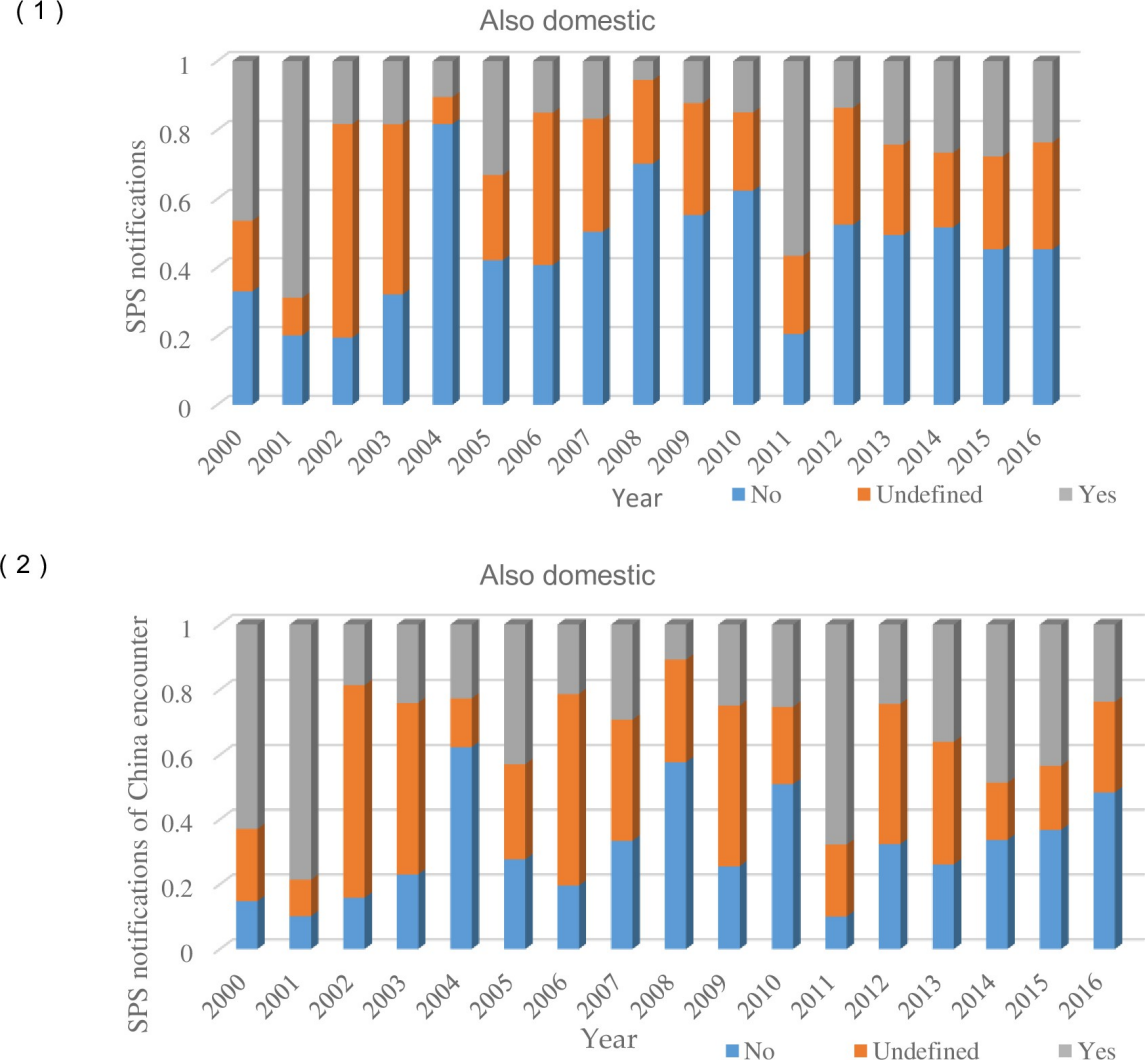
The SPS notifications (also domestic).

As for whether SPS measures are also applicable to domestic or not, SPS measures can be divided into three kinds, e.g., “yes”, “no” and “undefined”. Fig 3 draws the average number of "not also domestic" (45.39%), whose proportion is much higher than that of "also domestic" (28.41%) and "undefined"(26.19%). Accounting to the total number of notifications from 2000 to 2016, the number of “not also domestic" fluctuates from 33.04% in 2000 to a small peak (70.22%) in 2008, and then gradually dropped to 45.22% in 2015. Similarly, in terms of SPS measures encountered by China, more than half of the SPS measures are not equally applicable to the implementing country. Therefore, in the long run, under the global protectionism environment of "double standards", SPS measures are neither conducive to the sustainable development of high value-added international agricultural trade, or helpful to trade liberalization.

### The pertinence of SPS measures

**Fig 4:**
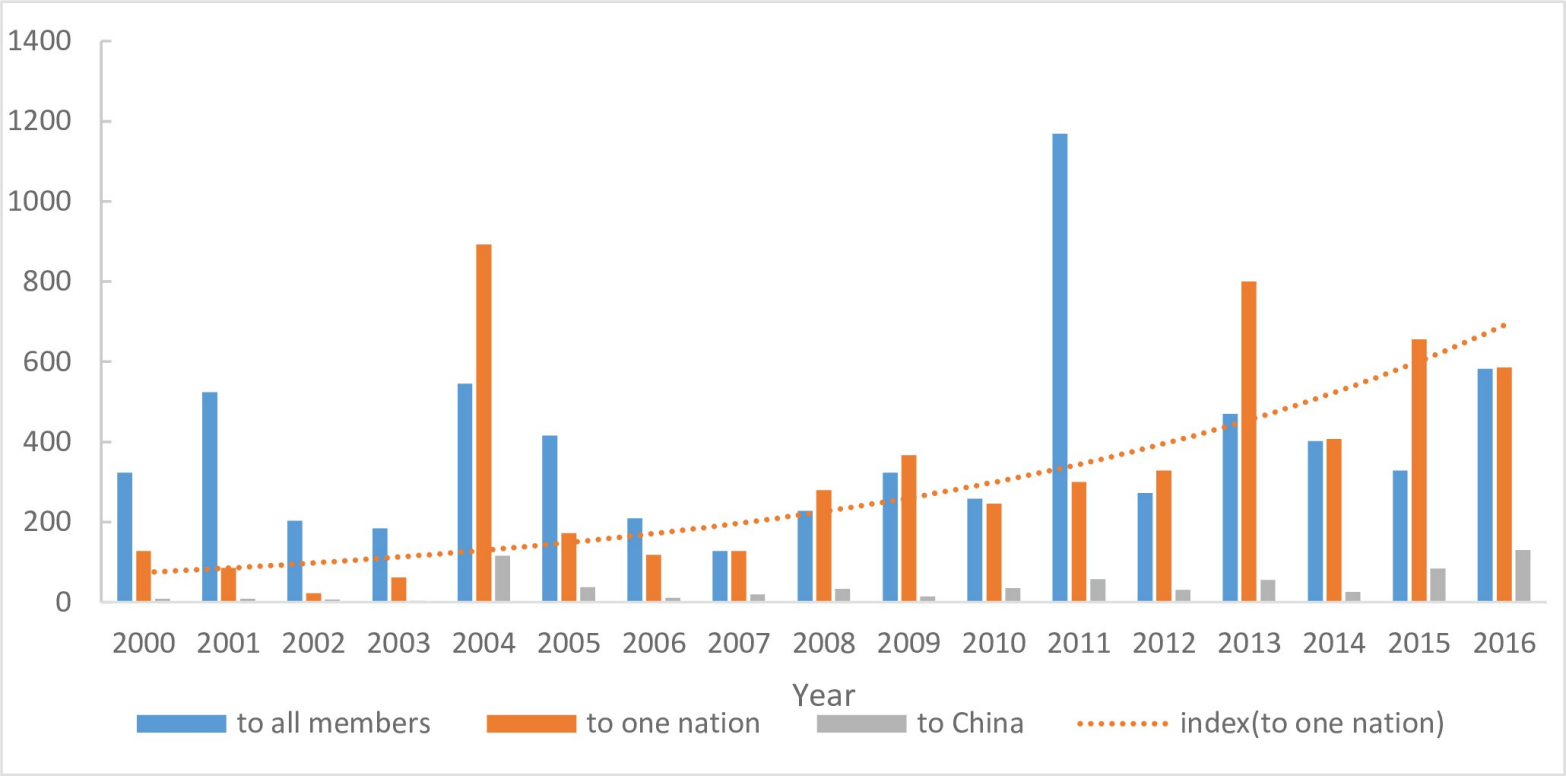
The SPS notifications China has encountered.

Fig 4 draws the pertinence of SPS measures (2000–2016). The number of “to all members” reaches a small peak in 2011 (1,169) and then remains at an average level of 346 cases. The proportion of SPS notifications of “to all members” is 6.5% higher than that of “to one nation”(46.69%), which is still very high. The number of “to China” accounts for 8.3% of the total SPS measures, but the number of SPS notifications for China continues to rise from 9 in 2000 to 130 in 2016, and the proportion of “only to China” is 8.3%. The absolutely rising trend of “to one nation” reflects the deteriorating market environment in the global agricultural markets.

### The diversity of SPS measures

By combining the number of SPS notification (Category A) notified by UNCTAD-Trains with the detailed names of SPS measures notified by WITS (World Integrated Trade Solution). S1 Table shows the proportion of different SPS categories. The SPS measures involve 8 categories and over 30 specific measures. A83, A22, A31, A14, A84, A33, A69 and A64 account 52.61% of the total number of notifications. When talking about China, A22, A83, A31, A14, A22, A84, A33 and A64 account 58.54% of the total number of notifications encountered by China.

## China’s agri-products export growth

### Export growth and product quality at national level

**Fig 5:**
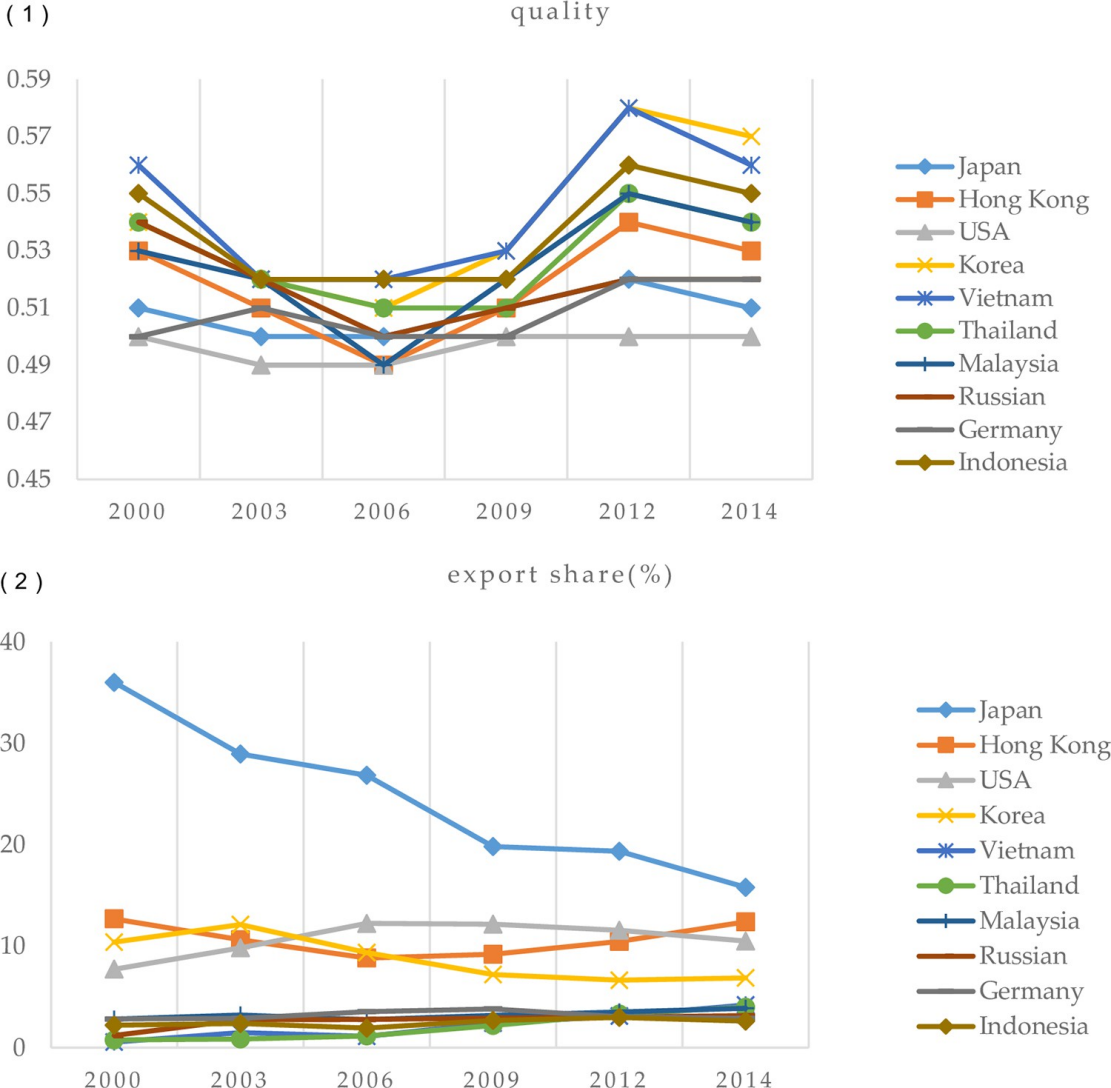
Heterogeneous export performance across destinations (2000–2014). Notes: Authors’ estimation using Gerneral Adiministration of Customs of the People’s Republic of China (GACC).

Fig 5 shows the growth performance across destinations China export mainly, including export value share and product quality. By comparing the growth rate of shared reallocation and quality level in different destinations, we find: (1) There are significantly different trends of export value share and export quality. (2) There is a sharp rise in the export quality level of Korea, Japan and Hong Kong when the growth rate of export value share is negative. Conversely, an obvious rise occurs in the export quality level of Thailand, Indonesia and Vietnam when the growth rate of export share is positive. (3) However, in some developed destinations, there are declined agri-products export quality level and a positive export shares growth in China, such as US and Russia. These different growth trends indicate the incoordination between export value share and product quality, and the complexity of export reallocation. So, from the perspective of multi-destination to study export growth is based on Chinese trade facts.

### Changes in the destinations at firm-level

Table 1 shows the number of destinations China export agri-products at firm-level. There is a relatively concentrated trend from 2000 to 2014 which present an obviously skewed characteristic of the distribution of destinations number. The firms that export to a single destination account for 63.2% of the total number of firms but only 25.6% of the export value. In contrast, firms that export to more than 6 destinations account for 8.8% of firms but 49.9% of value.

**Table 1:** Number of destinations China’s export agri-products (2000–2014).

**Number of Destinations**	**All the Countries**	**Except the Intermediaries**
**mean**	2.65	2.83
**min**	1	1
**median**	1.33	1.50
**max**	116.50	116.50
**Number of Destinations**	**Export Firm Ratio**	**Export Value Ratio**
**1**	63.18%	25.61%
**2–5**	28.00%	24.53%
**6–10**	5.51%	17.09%
**11–25**	2.87%	22.78%
**Above 25**	0.44%	9.99%

Sources: Authors’ estimation by using GACC.

In fact, there is a distinct dynamic characteristic in China’s agricultural export firms’ behaviors. Table 2 is the matrix depicting the conversion of destinations number in two adjacent periods. As we can see, the value on non-diagonal is the proportion of firms that changed destinations number, while the value on diagonal is the proportion of firms that remained unchanged in export destinations number. When comparing with the value on non-diagonal, the value on diagonal is significantly higher, which means the proportion of firms that remained unchanged in export destinations number is higher. What’s more, the value on non-diagonal is normal behaviors, which means there is a higher probability of switching destinations in China’s export.

**Table 2:** Destinations number conversion matrix of China agri-products firms export.

	Number (n) of Destinations in t-1					
**Number (N) of Destinations in t**	(t-1)/t	1	2–5	6–10	11–25	Above 25
	1	67.29%	21.08%	3.20%	1.04%	0.33%
	2–5	27.17%	67.06%	30.09%	4.98%	0.93%
	6–10	3.68%	10.44%	51.40%	20.03%	1.82%
	11–25	1.60%	1.39%	15.12%	68.93%	20.35%
	Above 25	0.26%	0.04%	0.20%	5.02%	76.57%
**The percent of firms exporting to n countries in the t-1 year**		39.86%	17.82%	37.96%	3.67%	0.69%

Notes: Each value is the annual average. In the matrix, x firms exported in categories in agri-products in the t-1 year, and y firms exported N categories agri-products in the t year. So, the conversion rate from n in t-1 year to N in the t year is y/x. Source: Authors’ estimation by using GACC (2017).

## Empirical framework and data

### Empirical framework

According to the asymmetric heterogeneous firm trade model (MO model) integrating the multi-destination [22], our empirical model chooses the improved gravity model. Chaney (2008) explained the definition of extensive and intensive margins [23], which give us a great reference of the empirical model and decomposed the export value share into extensive margin and intensive margin. The intensive margin can be further decompose into price index and quantity index. So, based on the improved gravity regression equation [23, 24], the empirical equation can be shown as:

$$lnQ_{lm}^{h}=\alpha_{0}+\alpha_{1}lnY_{m}+\alpha_{2}ln\varphi_{m}+\alpha_{3}ln\tau_{lm}+\alpha_{4}lnf_{lm}+\alpha_{5}ln\theta_{m}+\alpha_{6}\Theta +\epsilon$$ (1)

Where $Q_{lm}^{h}$ represent growth margins or quality upgrading of product *h* exported from country *l* to country *m*. *Y*_*m*_ is total income spent by workers in country *m*; φ_m_ is productivity in country *m*. *τ*_*lm*_, *f*_*lm*_ and *θ*_*m*_ respectively represent the variable costs (including SPS measures), fixed costs and the multilateral resistance; Θ means the other control variables, while the residuals are represented by *ϵ*.

### Data

1. Growth margins. Following Hummels and Klenow [25] and Bingzhan Shi [26], we measure the extensive margin, intensive margin, price index, and quantity index and we move the method to S1 Appendix. The HS6-digital country-product level data comes from UN Comtrade. It includes the data of China exports to 50 main destinations from 2000 to 2014.
2. Product quality. Following Demand Residual Method [6], we use HS8-digital firm level data to measure, and we move it to S2 Appendix. The HS8-digital firm level data comes from GACC (General Adiministation of Customs of the People’s Republic of China). It includes the data Chinese firms export to 50 destinations from 2000 to 2014. We use the logarithm of the quality difference between t and t-1 as the extent of quality upgrading.
3. SPS measures. The data is from the UNCTAD-Trains database and the WTO-SPS measures notification system. The former shows different SPS categories that is used to make benchmark regression. The latter includes more comprehensive data that is used to make in-depth analyses and robust regression. Because the new notifications are implemented within 6–9 months, there is a time lag for exporters to improve product quality. So, we take the lag notifications as SPS measures.
4. Multinational resistance (*MR*). Drawing on the definition of multinational resistance [27], if country *r* switch to export to countries other than *d*: ${MR}_{\gamma }^{-d}=\sum_{d=1}^{D}\theta_{d}^{-\gamma }$, where $\theta_{d}^{-\gamma }=\sum_{r=1}^{R}\frac{Y_{r}}{Y_{all}}\phi_{rd}$. *ϕ*_*rd*_ means the bilateral trade freedom if country *r* export to country *d*. $\theta_{d}^{-\gamma }$ means the bilateral resistance if countries other than *r* export to country *d*. [28]. *Y* means the GDP. Since the switch costs measure the trade resistance index from other countries, the larger the resistance from other countries, the more quality upgrading on original destination.
5. Fixed cost. If the degree of intervention in an importing country is smaller, the country will have a higher openness degree. (HFI). Its reciprocal is taken as the fixed trade cost, which is from the Heritage Foundation Inc.
6. Trade distance. The quality and safety of agri-products are affected by the “distance dilemma”, which is well-known as “Washington Apple” effect [29]. The higher product quality, the further destination it can be sold. The trade distance data is from CEPII Trade Prod database.
7. National agri-product production index. The higher national agri-products production index, the higher quality standards on import products. The data is from Food and Agriculture Organization of the United Nations (FAOSTAT).
8. Total income (GDP per capita). The total income of import country has a positive effect on the import products’ quality [30]. We can get the per capital GDP from the CEPII Gravity database.
9. Market concentration (*MC*). In $MC=\sum_{d=1}^{D}{\left(x_{d}/x\right)}^{2}$, *x* and *d* represent the value and destination of the exporter separately. The data is from GACC.
10. Diversification and Multination. Because the extensive margin is the impetus of the export growth, so the diversification in category and the multination in destination will affect the exporter’s decision. Exporter can upgrade the quality or export more categories in low quality standard or switch to low standard destinations [31]. The data is from GACC.

In addition, the regional economic integration (*FTA-WTO*), tariff barriers (ln(1+tariff)), the land area *ln(Area_agri)*, etc. are also been used as the controlled variable, which are from CEPII Gravity database. Descriptive statistics are shown in Table 3.

**Table 3:** Summary statistics.

Variables	N	Mean	Sd	Min	P50	Max
**SPS**	13343	0.58	2.54	0.00	0.00	71.00
**Extensive margin**	17632	0.52	0.37	0.00	0.57	1.00
**Intensive margin**	17632	0.09	0.16	0.00	0.03	1.00
**Price margin**	17632	0.83	0.50	0.00	0.92	1.99
**Quantity margin**	17632	0.08	0.15	0.00	0.02	1.00
**Quality(log)**	17632	2.12	1.37	0.00	2.04	4.61
**Fixed cost(log)**	13002	0.12	0.02	0.10	0.12	0.29
**Distance(log)**	13329	8.83	0.60	7.06	8.96	9.85
**MR (log)**	16572	0.87	0.59	0.05	0.72	2.61
**Production Index**	13329	108.79	13.73	87.28	111.72	129.10
**GDP per capita**	13329	23066.18	18719.74	350.29	20409.00	87998.50
**Area-agri**	13329	1151.62	3245.96	0.12	362.60	23782.18
**Multination**	17632	44.6	7.01	12.00	47.00	50.00
**MC**	15288	0.02	0.06	0.00	0.00	0.85
**Diversification**	15288	10.58	9.34	1.00	8.00	67.00
**Tariff(log)**	8282	1.89	1.28	0.00	1.95	7.48
**FTA-WTO**	13329	0.13	0.33	0.00	0.00	1.00

## Results

### The effects of SPS measures on growth margins and quality ugrading

Considering the possible problems of insufficient sample selection, heteroscedasticity, excessive dispersion and excessive frequency of zero data in the gravity model regression, PPML (Poisson pseudo-maximum-likelihood) regression can obtain the consistent estimators [32].

The specific results are shown in Table 4. Columns (1)-(6) respectively examine the effects of SPS measures on extensive margin, intensive margin, quantity index, price index, product quality and the extent of quality upgrading. SPS measures, as the quality upgrading compliance costs, have an inhibitory effect on extensive margin, intensive margin, quantity index and quality upgrading. First, the results not only indicate that the varieties and value of Chinese exports decrease under the influence of the SPS measures and but also well explain the "barrier" effect of SPS measures on the export growth directly. Second, SPS measures have a motivational effect on product quality and an inconspicuous effect on price margin, which reflect the positive effect of SPS measure on product quality, will the positive effect cannot increase the product price. It means measuring the quality directly instead of using price index as the proxy variable is necessary. This is consistent with the arguments that price index is not only affected by the product quality, but also affected by the export strategy [33, 34]. At last, when talking about the quality upgrading, export firms need to weigh compliance costs of SPS measures and switch costs of exporting to lower-standard countries. The SPS measures and multinational resistance cause these two kinds of cost. Columns (6) shows that the compliance costs of SPS measures negatively correlated with quality upgrading. A one-unit increase in SPS levels decreases quality upgrading by 44.4%; however, a one-unit increase in levels of multinational resistance increases the quality upgrading by 14.2%.

**Table 4:** The regression results of growth margins and quality upgrading.

	(1)	(2)	(3)	(4)	(5)	(6)
	Extensive margin	Intensive margin	Quantity margin	Price margin	Quality	Quality upgrading
SPS	-0.120	-2.557***	-2.277***	-0.119	0.149***	-0.444***
	(0.095)	(0.347)	(0.336)	(0.083)	(0.044)	(-0.122)
lnMR	0.085***	-0.096	-0.078	0.067***	-0.051***	0.142
	(0.016)	(0.053)	(0.049)	(0.013)	(0.006)	(-0.094)
lnFixedcost	-0.223***	-0.589***	-0.655***	0.046	0.015	0.291
	(0.054)	(0.169)	(0.176)	(0.041)	(0.018)	(-0.211)
Ln(Production index)	-0.036*	-0.133*	-0.120*	0.012	0.037***	-0.732***
	(0.018)	(0.060)	(0.056)	(0.017)	(0.008)	(-0.119)
Ln(GDP per capita)	0.008	-0.101***	-0.125***	-0.002	0.034***	-0.180***
	(0.008)	(0.021)	(0.021)	(0.007)	(0.003)	(-0.041)
MC	0.413***	1.725***	1.669***	0.043	0.134***	-2.090***
	(0.040)	(0.123)	(0.123)	(0.049)	(0.023)	(-0.437)
Diversification	0.817***	1.233***	1.792***	0.088*	-0.049**	-2.205***
	(0.037)	(0.143)	(0.124)	(0.038)	(0.019)	(-0.47)
Multination	2.478***	0.054	1.736**	2.182***	0.043	-3.883***
	(0.175)	(0.478)	(0.646)	(0.195)	(0.064)	(-0.499)
Ln(1+tariff)	-0.055***	0.021	0.018	-0.032***	-0.005**	0.015
	(0.005)	(0.015)	(0.013)	(0.004)	(0.002)	(-0.027)
lnDistance	-0.132***	-0.203***	-0.307***	0.018**	-0.028***	-0.007
	(0.007)	(0.021)	(0.021)	(0.006)	(0.003)	(-0.036)
Ln(Area-agri)	0.010	-0.276***	-0.237***	0.030	-0.065***	1.039***
	(0.020)	(0.067)	(0.062)	(0.016)	(0.007)	(-0.074)
FTA-WTO	0.068***	0.113**	0.129***	0.001	0.016**	-0.575***
	(0.014)	(0.037)	(0.036)	(0.012)	(0.005)	(-0.084)
Cons	-1.059***	0.651	0.981*	-1.216***	-0.695***	-3.900***
	(0.145)	(0.457)	(0.467)	(0.127)	(0.054)	(-0.607)
Obs	8015	8015	8015	8015	8015	8015
R2	0.247	0.094	0.164	0.075	0.056	0.078

Notes: The values in brackets are standard deviations

***, ** and * respectively mean statistical significance of 1%, 5% and 10%. All models are estimated using PPML. SPS measure is from UNCTAD-Trains.

### Heterogeneity across country different trade routes

Most of the SPS measures comes from the developed countries and their standards is stricter than in the developing countries. Therefore, distinguishing the heterogeneity across country different trade routes is indispensable. We divide the export destinations into developed countries and developing countries, and then we examine the effect of SPS measures on the quality upgrading separately. Table 5 shows the results of developed and developing countries’ SPS measures. We find: (1) wherever China export, the SPS measures have a negative effect on the quality upgrading. (2) When controlling for other influence factors, if China exports agri-products to developed countries, a one-unit increase in compliance costs reduces the extent of quality upgrading by 35.1%. But, if China exports agri-products to developing countries, a one-unit increase in compliance costs reduces the extent of quality upgrading by 22.4%. The negative effect of compliance costs from the developed countries is greater. (3) Meanwhile, if China exports agri-products to developed countries, a one-unit increase in levels of switch costs increases the extent of quality upgrading by 14.1%. But, if China exports agri-products to developing countries, a one-unit increase in levels of switch costs increases the extent of quality upgrading by only 1.9%. The positive effect of switch costs from the developed countries is greater. It reflects that some firms prefer to bear the switch costs than to upgrade products’ quality, so they can pursue the profit maximization in the short term by shift the export path to the developing countries (low quality standards).

**Table 5:** Regression results of developed and developing countries.

Quality upgrading		
	developed	developing
**SPS**	-0.351***	-0.224**
	(0.089)	(0.079)
**LnMR**	0.141***	0.019***
	(0.013)	(0.004)
**Other variables**	control	control
**Cons**	-2.296***	0.285*
	(0.355)	(0.133)
**Observations**	2381	9325
**R2**	0.331	0.298
**Estimator**	PPML	PPML

Notes: The values in brackets are standard deviations

***, ** and * respectively mean statistical significance of 1%, 5% and 10%. SPS measure is from WTO-SPS.

### Heterogeneity across firm different trade routes

When talking about the reasons for quality upgrading, it depends on both their motivations and upgrading abilities. We introduce the distance to the frontier model in this part. Based on the ABGHP model [35], the frontier model presents the leaders have a higher ability to upgrade. Here now, we assume the agricultural leaders’ quality is closed to 1.

Table 6 reports the regression results of technology leaders and technology laggards. The empirical results (Column 6) shows that: (1) the marginal impacts of compliance costs and switch costs on quality upgrading are in line with the general regression. (2) Row 3 show the results of integrating the compliance costs with distance. The quality upgrading is attenuated. It also indicates that the negative effect of compliance costs on theleaders is less than for the laggards by 8.2%. (3) Row 5 shows the results of interacting the switch costs with front distance. The quality upgrading is magnified. It indicates that the switch costs promotes the leaders more than the laggards by 2.8%. (4) Row 6 shows the results of interacting compliance costs, switch costs and the distance. It indicates that after comprehensively considering the compliance costs and switch costs, the laggards are more inclined to refuse upgrading by 1.1%.

**Table 6:** Regression results of technology leaders and technology laggards.

Quality upgrading			
**SPS**	-0.304**	-0.161***	-0.602***
	(0.094)	(0.027)	(0.094)
**Front Distance**	0.085***	0.168***	0.163***
	(0.001)	(0.002)	(0.002)
SPS* Front Distance	0.019		0.082***
	(0.016)		(0.016)
**LnMR**	0.054***	0.207***	0.208***
	(0.001)	(0.003)	(0.003)
LnMR * Front Distance		-0.028***	-0.028***
		(0.000)	(0.001)
SPS * LnMR * Front Distance			-0.011***
			(0.002)
**Other variables**	control	control	control
**Cons**	0.423***	-0.015	0.005
	(0.025)	(0.026)	(0.026)
**N**	204355	204355	204355
**R-Sq**	0.092	0.112	0.113
**Extimator**	PPML	PPML	PPML

Notes: The values in brackets are standard deviations

***, ** and * respectively mean statistical significance of 1%, 5% and 10%. SPS measure is from WTO-SPS.

### Robust analysis

We make the robustness test from aspects of the empirical regression method and sample size selection (see Table 7 (1)-(3)). Firstly, in column (1), we use the Tobit to check the robust regression that can effectively deal with the censored data issue as well as the heteroscedasticity in the error terms [32]. Secondly, in column (2), considering trade intermediaries are not effected by market trade costs, but agri-food processors do. So, we leave out the trade intermediaries. Finally, in column (3), we chose the labor-intensive agri-products as the samples, which are the main export agri-products in China, because the China Agricultural Trade Development Report (2017) shows the export proportion of China’s labor-intensive agri-products in 2016 is 67%.

**Table 7:** Robust regression results.

	(1)	(2)	(3)
**SPS**	-0.525***	-0.499***	-0.838**
	(0.070)	(0.069)	(0.277)
**Ln MR**	0.071***	0.056***	0.095***
	(0.009)	(0.009)	(0.022)
**Ln(Fixed cost)**	0.046**	0.020	0.009
	(0.017)	(0.017)	(0.041)
**Ln (Production Index)**	2.396***	3.059***	1.690***
	(0.179)	(0.175)	(0.440)
**Ln(GDP per capita)**	-0.144***	-0.128***	-0.176***
	(0.010)	(0.010)	(0.025)
**MC**	0.498***	0.455***	1.164***
	(0.134)	(0.130)	(0.350)
**Diversification**	-1.961***	-1.978***	-1.970***
	(0.183)	(0.188)	(0.329)
**Multination**	-0.141***	-0.140***	-0.171***
	(0.005)	(0.005)	(0.018)
**Ln(1+tariff)**	0.072***	0.100***	0.065
	(0.015)	(0.015)	(0.037)
**Ln(Distance)**	0.170***	0.164***	-0.070
	(0.029)	(0.029)	(0.073)
**Ln(Area-agri)**	-0.016*	-0.022**	0.000
	(0.008)	(0.008)	(0.019)
**FTA-WTO**	-0.406***	-0.466***	-0.359**
	(0.051)	(0.049)	(0.123)
**Cons**	0.325	-0.090	2.779***
	(0.307)	(0.304)	(0.809)
**Observations**	11706	11437	2278
	**Estimatior**	Tobit	Tobit	Tobit

Notes: The values in brackets are standard deviations

***, ** and * respectively mean statistical significance of 1%, 5% and 10%. SPS measure is from WTO-SPS.

## Discussion

Our empirical findings can be broadly categorized into three sets. First, we show that an increase in the notification of SPS measures in an importing country limits agricultural export growth by reducing the varieties of commodities traded, the value of commodities traded, the quantity and price of commodities traded. These findings are consistent with existing works on SPS/TBTs measure [8, 36, 37] and existing works on import standards [38]. SPS measures play as the cost-added trade barriers, which will raise the variable trade cost and fixed trade cost, and induce the selection effect [2, 24, 39].

Second, we show that an increase in the notification of SPS measures in an importing country incentive agricultural export quality level, but limits the extent of quality upgrading. What’s more, SPS measures implemented in developed countries and developing countries have different trade effect. This is consistent with the findings that stricter standards in a given destination has the price raising effect and null quality-upgrading effect for South-North trade [4]. When dealing with the increasing SPS measures, China’s export quality-upgrading is inconspicuous and we address some reasons as follows.(1) there is not a sufficient ability to upgrade quality. In the global agri-product value chain (AVC), China mostly plays as the “raw material supplier” or “production processor” [40–42]. There is still great room for improvement (e.g. modernization and mechanization) in the future. (2) there is a lower investment return rate of agri-products’ quality upgrading [43, 44]. Especially, when comparing with non-agricultural industries, agri-products take longer production cycle and more difficult technologies. (3) the impact of Chinese trade policy is ambiguous. Chinese trade policy is export-oriented which is made solely based on the “quantity dimension”, but ignores the importance of quality upgrading. There is no significant difference exists in the export subsidies or export tax rebates, no matter the firms export to the high-standard markets or low-standard markets. This policy will not incentive the exporters to upgrade. Therefore, in view of heterogeneous SPS measures in the international market, even if some importers enhance quality threshold, the export impetus of short-term profit maximization will distort the incentive effect of the SPS measures.

Finally, we show that exporters will consider to upgrade products’ quality or not depending on compliance costs and switch costs. Actually, if and only if the exporters choseto upgrade quality, the higher SPS measures have a positive effect on quality upgrading. But if the exporter chose to switch export markets oppositely, exporters can keep export growth without upgrading quality level in the short term. This is consistant with Fontagné & Orefice [39]. When dealing with the enhancement of heterogeneous SPS measures, the leaders have a stronger capability to upgrade and keep in line with enhanced quality standards, while the laggards have weak upgrading capabilities, and the compliance costs has a negative impact on quality upgrading and "learning from doing" effect [45]. These results are based on the multinational trade model, which cannot be explored under the bilateral trade model, where we find the quality standards improve the export quality level.

For policy makers, (i) Export firms need to be aware of the barriers and harms of international SPS measures and actively build the development momentum in the long term. For example, try to improve the regional brand building of agri-products and the corporate social responsibility, and the incentive effect of non-institutional factors(e.g., moral trust); (ii) The government can start to stimulate food safety regulations, improve quality supervision systems and digital granting farmers. These internal mechanisms are playing important role in the development of quality upgrading. Meanwhile, a slight tilt to high-quality firms on export tax rebates and export subsidy policies will also enhance the trade effectiveness of policy interventions. (iii) It is recommended that the supply-side structural reform in China’s agriculture should ensure the enhancement of the domestic quality standards. China should aim at international standards to promote domestic technological transformation, revise China’s national food safety standards, and adhere to the concept of equal participation of developing countries in global economic governance, and form a global high-standard trade network.

Our study is not without limitations. Due to the limitation of the data, our estimation ignore the numbers of employees, technical level of labor force, total factor productivity at firm level. While the heterogeneity of SPS measures do not match to the HS6 digital accurately. In order to deal with data issue, case study method or a more detailed database (e.g. Homologa database) should be introduced. Future analyses should consider more seriously the possibility of export subsidy policy and the environment effect of SPS measures. In addition, further analyses can study the differences in SPS measures between China and the international high-standard countries. Try to figure out the problems existing in the domestic supply chain and global value chain is also necessary.

## Conclusions

This paper tries to answer the question “how the cross-country differences in quality standards affect the sustainable agricultural growth (e.g., extensive margin, intensive margin, quantity index, price index/product quality and quality upgrading) in China?” Based on the macro-trade countries-level data of UNCTAD-Trains from 2000–2014 and the micro-trade firm-level data of GACC from 2000–2014, we took into PPML empirical regression method. The results show that: (1) the incentive effect of SPS measures on growth impetus is significant because the heterogeneous quality threshold increase the product quality, and decrease the extensive margin, intensive margin and quantity index. (2) The mechanism of SPS measures on quality upgrading is inconspicuous in the long term. When facing the heterogeneous SPS measures in different destinations, exporters have to weigh the compliance costs and switch costs. Especially, the exporters choose to switch market and pursue the short-term profit maximization. It may cause the quality upgrading trap of China’s agri-product export. (3) Because of cross-country differences in SPS standards, standards from developed countries have less effective quality upgrading effect. (4) Because of cross-firm differences in the abilities to deal with SPS standards, the laggards firms have the higher probability to switch to lower entrance barrier countries. In summary, the laggards increase the possibility of falling into the quality upgrading trap.

## Supporting information

S1 Table The category and proportion of SPS measures encountered by the world and China. (DOCX)

S1 Appendix Measures of the extensive and intensive margins following Hummels & Klenow (2005) and Shi (2013). (DOCX)

S2 Appendix measures of the quality following Khandelwal et al., (2013). (DOCX)

S1 File (ZIP)
