# Supplementary material for: Effects of heterogeneous SPS measures on agricultural growth: Evidence from China
Source: PLoS One. 2022 May 10;17(5):e0266904. doi: 10.1371/journal.pone.0266904 (PMC9089914; doi:10.1371/journal.pone.0266904)
Supplement: S1 Table — (DOCX) [file pone.0266904.s001.docx]

Supporting information

S1 Table. The category and proportion of SPS standards encountered by the world and China

| **NTM Code** | **SPS category** | **World (%)** | **China (%)** |
| --- | --- | --- | --- |
| **A11** | Temporary geographic prohibitions for SPS reasons | 4.56 | 1.57 |
| **A12** | Geographical restrictions on eligibility | 0.45 | 0.44 |
| **A13** | Systems Approach | 0.88 | 0.74 |
| **A14** | Special Authorization requirement for SPS reasons | 6.02 | 6.49 |
| **A15** | Registration requirements for importers | 2.06 | 1.82 |
| **A19** | Prohibitions/restrictions of importsfor SPS reasons n.e.s. | 2.38 | 2.50 |
| **A21** | Tolerance limits for residues of or contamination by certain (non-microbiological) substances | 3.77 | 6.00 |
| **A22** | Restricted use of certain substances in foods and feeds and their contact materials | 7.14 | 11.78 |
| **A31** | Labelling requirements | 6.19 | 9.17 |
| **A32** | Marking requirements | 1.11 | 1.15 |
| **A33** | Packaging requirements | 5.08 | 5.30 |
| **A41** | Microbiological criteria of the final product | 3.09 | 4.46 |
| **A42** | Hygienic practices during production | 1.41 | 1.81 |
| **A49** | Hygienic requirements n.e.s. | 0.40 | 0.57 |
| **A51** | Cold/heat treatment | 2.88 | 2.00 |
| **A52** | Irradiation | 0.33 | 0.31 |
| **A53** | Fumigation | 2.37 | 1.37 |
| **A59** | Treatment for elimination of plant and animal pests and disease-causing organisms in the final product, n.e.s. | 1.71 | 1.41 |
| **A61** | Plant growth processes | 1.42 | 0.46 |
| **A62** | Animal raising or catching processes | 1.67 | 0.98 |
| **A63** | Food and feed processing | 3.01 | 3.19 |
| **A64** | Storage and transport conditions | 4.58 | 4.86 |
| **A69** | Other requirements on production or post-production processes, n.e.s | 4.82 | 2.81 |
| **A81** | Product registration requirement | 1.03 | 1.65 |
| **A82** | Testing requirement | 3.83 | 4.66 |
| **A83** | Certification requirement | 13.51 | 9.58 |
| **A84** | Inspection requirement | 5.29 | 5.36 |
| **A85** | Traceability requirements | 4.00 | 3.05 |
| **A86** | Quarantine requirement | 3.74 | 2.97 |
| **A89** | Conformity assessment related to SPS n.e.s. | 0.90 | 1.10 |
| **A9** | SPS standards n.e.s. | 0.37 | 0.43 |

Notes: Data from UNCTAD-Trains
