# Supplementary material for: Effects of heterogeneous SPS measures on agricultural growth: Evidence from China
Source: PLoS One. 2022 May 10;17(5):e0266904. doi: 10.1371/journal.pone.0266904 (PMC9089914; doi:10.1371/journal.pone.0266904)
Supplement: S1 Appendix — (DOCX) [file pone.0266904.s002.docx]

Supporting information

S1 Appendix: Measures of the extensive and intensive margins following Hummels & Klenow（2005）and Shi(2013)

Hummels and Klenow（2005）construct empirical counterparts to extensive margin (E), intensive margin (I), price index (P) and quantity index (Q). For the case when country c export product j is a subset of r export to the global market in period t, the export value share is defined as

$S_{Jt}=\frac{M_{\mathrm{ct}}}{M_{\mathrm{rt}}}=\frac{\sum_{j\in N_{\mathrm{ct}}} M_{\mathrm{cjt}}}{\sum_{j\in N_{\mathrm{rt}}} M_{\mathrm{rjt}}}=\frac{\sum_{j\in N_{\mathrm{ct}}} M_{\mathrm{rjt}}}{\sum_{j\in N_{\mathrm{rt}}} M_{\mathrm{rjt}}}*\frac{\sum_{j\in N_{\mathrm{ct}}} M_{\mathrm{cjt}}}{\sum_{j\in N_{\mathrm{ct}}} M_{\mathrm{rjt}}}=E_{\mathrm{Jt}}*I_{\mathrm{Jt}}$ （2）

$S_{Jt}$ represents the export value share in period t，$M_{ct}$ and $M_{rt}$ represent the export value of country c and r respectively. $M_{cjt}=p_{cjt}*x_{cjt}，M_{rjt}=p_{rjt}*x_{rjt}$. $N_{ct}$ and$N_{rt}$ represent the categories in country c and country r. $E_{Jt}$ measures the proportion of overlapping products of country c export in the world’s export. The larger $E_{Jt}$ is, the higher the overlap degree of export product categories of country c and the world is, and the greater the breadth of products. $I_{Jt}$measures the proportion of country c exports the overlapping products in the world's exports the overlapping products. The larger $I_{Jt}$ is, the more c exports in the overlapping products, and the greater the depth of product.

On the basis of binary margins, the three dimensions of export growth are further decomposed into extensive margin, price index and quantity index:

$S_{Jt}=E_{\mathrm{Jt}}*I_{\mathrm{Jt}}=E_{\mathrm{Jt}}*P_{\mathrm{Jt}}*X_{\mathrm{Jt}}$ (3)

$P_{\mathrm{Jt}}=\prod_{j\in N_{\mathrm{ct}}} {({p_{\mathrm{cjt}}}/{p_{\mathrm{rjt}}})}^{\omega_{\mathrm{jt}}}$ (4)

$X_{\mathrm{Jt}}=\prod_{j\in N_{\mathrm{ct}}} {({x_{\mathrm{cjt}}}/{x_{\mathrm{rjt}}})}^{\omega_{\mathrm{jt}}}$ (5)

In the ratio, $\omega_{jt}=\frac{\left( \frac{\emptyset_{cjt}-\emptyset_{rjt}}{{ln\emptyset}_{cjt}-{ln\emptyset}_{rjt}} \right)}{\sum_{j\in N_{ct}} \frac{\emptyset_{cjt}-\emptyset_{rjt}}{{ln\emptyset}_{cjt}-{ln\emptyset}_{rjt}}}$ , $\emptyset_{cjt}=\frac{p_{cjt}*x_{cjt}}{\sum_{j\in N_{ct}} p_{cjt}*x_{cjt}}$ and$\emptyset_{rjt}=\frac{p_{rjt}*x_{rjt}}{\sum_{j\in N_{ct}} p_{rjt}*x_{rjt}}$ represent the value share of product j in country c and r.
