# Supplementary material for: Effects of heterogeneous SPS measures on agricultural growth: Evidence from China
Source: PLoS One. 2022 May 10;17(5):e0266904. doi: 10.1371/journal.pone.0266904 (PMC9089914; doi:10.1371/journal.pone.0266904)
Supplement: S2 Appendix — (DOCX) [file pone.0266904.s003.docx]

Supporting information

S2 Appendix: measures of the quality following Khandelwal et al., (2013)

Khandelwal et al., (2013) uses the DSM quality measurement model. The premise is that when the product prices are the same, a product with a higher market share represents a higher quality. Assume that the consumer's CES utility function is:

$U=\left[ \int_{h\in H} \left[ \varphi(h)q(h) \right]^{(\sigma-1)/\sigma}d_{h} \right]^{\sigma/(\sigma-1)}$ (6)

In the Eq (6), *q(h)* and *φ(h)* denote the quantity and quality of h, respectively, and σ represents the elasticity of substitution among different varieties of products in the same category. The budget constraint function for consumers in country c to purchase product h is as follows:

$q_{\mathrm{ocht}}=\left( \varphi_{\mathrm{ocht}} \right)^{\sigma-1}{(p_{\mathrm{ocht}})}^{-\sigma}P_{\mathrm{ct}}^{-1}Y_{\mathrm{ct}}$ (7)

In the Eq (7),$q_{ocht}$,$p_{ocht}$, and $\varphi_{ocht}$ represent the quantity, price, and quality level of the products of country c, consumer n, and country h of product t in period t; $P_{ct}$ represents the price index of importing country at period t. $Y_{ct}$ represents the income level of importing country in period t. Take the natural logarithm to the Eq (7), we can sort it out and get the following regression equation, which is the core equation for measuring quality:

$\ln q_{\mathrm{ocht}}+\sigma lnp_{\mathrm{ocht}}=\alpha_{h}+\alpha_{\mathrm{ct}}+e_{\mathrm{ocht}}$ (8)

In the Eq (8),$\alpha_{h}$indicates the fixed effect of the product, which can effectively control the impact of product-level variables on product quality. $\alpha_{ct}$ indicates the country-time two-dimensional virtual variable. It can effectively control variables (e.g., the gross domestic product of exporting countries) that change simultaneously with the exporting country and time. $e_{ocht}$ represents a residual term that includes product quality. We can get $e_{ocht}$ at each product level through regression. The calculation equation of quality is as follows:

$\mathrm{Quality}_{ocht}=\ln\left( \hat{\varphi}_{\mathrm{ocht}} \right)=\frac{\hat{e}_{ocht}}{\hat{\sigma}-1}$ (9)

In order to facilitate the comparison of the quality in different countries and obtain the overall quality level, it is inevitable to standardize the quality as follows:

$\hat{\mathrm{quality}}=\frac{{quality}_{ocht}-{quality}_{min}}{{quality}_{max}-{quality}_{min}}$ (10)

${quality}_{min}$and ${quality}_{max}$ respectively represent the minimum and maximum quality level of all agricultural exporting countries in a certain year for the HS6 digital level.$\hat{\mathrm{quality}}\in$[0,1].
