# Supplementary material for: Effects of heterogeneous SPS measures on agricultural growth: Evidence from China
Source: PLoS One. 2022 May 10;17(5):e0266904. doi: 10.1371/journal.pone.0266904 (PMC9089914; doi:10.1371/journal.pone.0266904)
Supplement: S1 File — (ZIP) [file pone.0266904.s004.zip › supporting information-ZIP file archive/data availability.docx]

Data availability statement

**There are legal restrictions to sharing my data publicly.** Data is contained within the article can be divided into three parts. **The first** **part** is available from UN COMTRADE database (<https://comtrade.un.org>), which is used to measure the export growth margins. We tried to upload the data but failed because the data is too large (13G) for validation. So, it is advised to download the data from the <https://comtrade.un.org>. “Trade Data” includes some variables (reporter, partner, year, commodity code, trade direction, trade value, trade quantity, trade weight) and they are applicable for anyone. The second part is available from UNCTAD-TRAINS database (<https://trainsonline.unctad.org/detailedSearch>), which is used to present the SPS measures. It can be downloaded from the “other” section. We can share the first two parts data publicly, except the third part. Because the third part data is from the third party who bought from Gerneral Adiministration of Customs of the People’s Republic of China (GACC), and authors do not have permission to share the data. Others can connect to the third part named “ Beijing Soz Data Technology Co., Ltd” by email([service@EPSChinaData.com](mailto:service@EPSChinaData.com)) or the website ([www.epsnet.com.cn](http://www.epsnet.com.cn) ). Any institution can get the GACC data through this company. ( Company profile: Beijing Soz Data Technology Co., Ltd., established in 2017 and headquartered in Haidian District, Beijing, is a company of high and new technology. Focusing on data services, software development, and information consultancy, we are a professional provider of data services, product, and project solutions, and we are committed to providing our users with easy, professional, and superior data solutions. Ever since our establishment, we have been taking data as our basis, technology development as pilot and market as our guidance, highly attaching importance to the input of technology and data storage, and devoting ourselves to reconstructing a new data service model by way of technology development. Our products, China Microeconomic Data Query System and EPS China Data, have been well received by our users. In the meanwhile, we keep market and users in mind. Our proprietary development, Contest Participation Cloud System, is available, free-of-charge, to many higher institutions and over 10,000 students for their examination and contests. Besides, we also take part in off-line activities of “contest and knowledge for learning”.)
